# Changes of Dry Eye Parameters Especially Meibomian Gland Functions After Punctal Plugs Insertion in Aqueous-Deficient Dry Eye Patients

**DOI:** 10.3389/fmed.2022.849700

**Published:** 2022-03-02

**Authors:** Tingting Liu, Shulin Liu, Meiqi Gan, Yuqin He, Hongxue Fu, Mei Xu

**Affiliations:** Chongqing Key Lab of Ophthalmology, Chongqing Eye Institute, Chongqing Branch of National Clinical Research Center for Ocular Diseases, The First Affiliated Hospital of Chongqing Medical University, Chongqing, China

**Keywords:** Sjögren's syndrome, dry eye, meibomian gland, punctal and canalicular obstruction, Smart Plug

## Abstract

**Background:**

The study aimed at investigating the changes of dry eye parameters, especially the meibomian gland function in dry eye patients with primary Sjögren's syndrome (SS) and non-Sjögren's syndrome (non-SS) before and after punctal plugs insertion.

**Material and Methods:**

The SS and non-SS dry eye patients that received punctal plugs insertion were prospectively investigated. This study recruited fifty patients. The standardized patient evaluation of eye dryness (SPEED), ocular surface disease index (OSDI), tear meniscus height (TMH), non-invasive Keratographic breakup time (NIKBUT), Schirmer test without anesthesia (Schirmer I Test, SI T), corneal fluorescein staining (CFS), the meibomian gland dropout (meiboscore), meibum expressibility score (MES), meibum quality score (MQS), lid margin abnormalities and the lipid layer thickness (LLT) were analyzed at pre-obstruction, 2 weeks, 2 months and 6 months following the obstruction.

**Results:**

Our study observed a statistically significant improvement in ocular symptom scores (SPEED and OSDI scores) after punctal plugs insertion at every visit in both SS and non-SS patients (all *p* < 0.05). A similar pattern was observed in TMH, SI T, NIKBUT and CFS score in both groups (all *p* < 0.05), except that NIKBUT and CFS score had no obvious change in SS group at 6 months (*P* > 0.05). In terms of the meibomian gland evaluation, meiboscore did not change significantly, MES decreased at 2 and 6 months and MQS decreased only at 2 months in both groups. The lid margin abnormalities of the non-SS group were significantly improved at 2 and 6 months, while that of the SS group had changes only at 2 months. Interestingly, LLT in non-SS group continued to rise, reaching a higher level at 2 months (*p* < 0.05), while LLT in SS group increased only at 2 months (*p* < 0.05). Meanwhile, after the puntcal plugs insertion, non-SS group showed better outcomes concerning some parameters, such as lower ocular symptom scores, higher TMH and significantly greater LLT, compared with that in SS group (all *p* < 0.05).

**Conclusions:**

Our study revealed that dry eye symptoms and signs, including meibomian gland function, improved for at least 6 months in non-SS dry eye patients as well as in SS dry eye patients after punctal plugs insertion.

## Background

Dry eye is one of the most common clinically observed ocular surface diseases (1). The TFOS DEWS II report confirmed tear hyperosmolarity, along with tear instability, as the core drivers of dry eye diseases (DED) (1, 2). Based on predominant etiology, two major subtypes of DED are defined, aqueous-deficient dry eye (ADDE) and evaporative dry eye (EDE) (1). Sjögren's syndrome (SS) is a chronic autoimmune disease exhibiting the feature of infiltration of salivary and lacrimal glands by activated T-cells which cause acinar and ductular cell death and hyposecretion of the tears or saliva (2, 3). Thus, dry eye correlated with SS is typically classified as ADDE (1). As for the EDE, meibomian gland dysfunction (MGD) is considered as the main cause (1). So the background of the two kinds of DED is different from the view of pathophysiological aspect of dry eye.

However, in recent years, it is reported that SS and MGD are related to each other and MGD participates in the pathogenesis of dry eye disease related to SS as well. Several studies have reported abnormal evaporation rates and clinically significant destruction of meibomian glands in SS patients (4–7).

Punctal obstruction is usually a non-pharmacological therapy for ADDE patients who had poor response to other treatment (8). It has already been proved to be effective in ADDE associated with SS patients (9, 10). In the previous study, many researches have focus on the changes of tear volume (the central portion of the lower tear meniscus radius, TMR) and precorneal tear film (TF) in ADDE after punctal obstruction. However, the changes in morphology and function of meibomian glands after applying punctal plugs in SS patients are still unclear.

Thus, the aim of this study is to evaluate associated changes in dry eye parameters following punctal obstruction in ADDE cases, especially the manifestation of MGD.

## Subjects and Methods

This prospective study was approved by the institutional review board of the First Affiliated Hospital of Chongqing Medical University and adhered to the tenets of the Declaration of Helsinki. All patients were fully informed of the details and possible risks of the procedure. Consents in written form were acquired from all participants prior to any procedure here.

### Subjects and Procedures

Primary SS ADDE patients between Jan 2019 to Oct 2020 at dry eye clinical center in the First Affiliated Hospital of Chongqing Medical University were included. Gender- and age-matched non-SS ADDE patients seen during the same period of time were set as control group. Primary SS patients all met the criteria reported by the American–European Consensus Group (11). ADDE was diagnosed as having decreased tear production ( ≤ 5 mm/5 min in Schirmer I test) etc. (1). Inclusion criteria also included that both SS and non-SS patients received preservative-free artificial tear (0.1% sodium hyaluronate) treatment for at least 2 months before this study and the dry eye symptoms were not relieved. Exclusion criteria included pregnant or lactating women, acute inflammation or infection of the eye, other ocular surface diseases, history of ocular surface surgery or lid abnormalities. All patients were diagnosed and evaluated by one ocular surface disease specialist (MX).

Polyacrylic acid punctal plugs (Smart Plug, Medennium, US) were inserted into the lower lacrimal duct in both eyes of all the included patients and preservative-free artificial tears (0.1% sodium hyaluronate) were permited to use 4 times a day. Punctal plugs remained *in-situ* and there had no obvious complications during the follow-up visits. The right eyes of both groups were chosen for the statistical analysis.

All subjects underwent a clinical evaluation at each visit (pre-punctal obstruction, 2 weeks, 2 months, and 6 months after punctal obstruction), which included ocular surface examination, meibomian gland evaluation and measurement of lipid layer thickness (LLT). Before clinical evaluations, patients were required to complete Ocular Surface Disease Index (OSDI) questionnaire and the Standard Patient Evaluation of Eye Dryness (SPEED). Data included age, gender was collected in both groups.

### OSDI and SPEED

Standard OSDI questionnaire was employed to evaluate the frequency of symptoms over the preceding weeks (12). Total OSDI score equals [(sum of the scores for each question answered) ×100]/[(overall number of questions answered) ×4] and the scores ranged from 0 to 100. The SPEED was used to evaluate frequency and severity of dry eye symptoms especially for the assessment of longer-term symptom changes over 3 months with a score ranging from 0 to 28. It should be noted that the higher the scores of either of the two questionnaires were, the more severe the symptoms were (13).

### Ocular Surface Examination

Ocular surface examination consisted of the measurement of tear meniscus height (TMH), test of non-invasive Keratograph break-up time (NIKBUT), Schirmer test without anesthesia (Schirmer I test, SI T) and corneal fluorescein staining (CFS). Ocular surface examination was performed 2 h after using artificial tears. Since forced eye opening required for the assessment of tear film stability increased TMH possibly due to reflex tear secretion, so TMH should be measured for accuracy before a non-invasive assessment of tear film stability (14).

TMH was measured using a keratograph 5M (Oculus GmbH, Wetzlar, Germany), which is equipped with a modified tear film scanning function. Lower tear film meniscus images were captured and TMH was measured before NIKBUT measurements in each subject.

NIKBUT was evaluated automatically through a Keratograph 5M. We required that the patients to blink first and subsequently open their eyes as long as possible. The time between the last blink and the first sign of distortion of the ring pattern was recorded. The process was repeated three times and the average of the three measurements was recorded as the final BUT.

SI T was performed with the patient's eyes closed for 5 min without anesthesia. The tear strip (30 mm; Jingming Tianjin, China) was folded in the front, positioned in the mid-lateral portion of the lower fornix for 5 min. The length of the wetting strip was recorded using the millimeter scale.

The score of CFS was evaluated through a white light and cobalt blue filter. The cornea was divided into four quadrants (superior nasal, inferior nasal, inferior temporal, and inferior temporal) and each quadrant got 0–3 points according to the degree of staining (15). Corneal staining was graded individually as follows: 0 point (no staining), 1 point (mild staining), 2 points (staining between 1 and 3), and 3 points (severe staining with bulk or strip staining). The total score of each eye was 0~12 points (15).

### Meibomian Gland Evaluation

Meibomian gland evaluation consisted of meibomian gland structure, gland morphology (gland dropout), gland function (meibum expressibility and quality) and lid margin assessment. We also measured lipid layer thickness (LLT) using the LipiView® interferometer (TearScience, Inc., Morrisville, NC) since ocular surface lipids are closely related to meibomian gland function.

The meibography images of the upper and lower eyelids were documented to assess the structure of the meibomian gland after satisfying focus using the LipiView® interferometer. The meibomian gland dropout (meiboscore) was analyzed by using Image J in Keratograph 5M as described previously by Pult and Nichols (16). The meibomian gland dropout rate was divided into four grades: grade 0 (no meibomian gland loss was detected), grade 1 (no more than 33% of meibomian gland loss was detected), grade 2 (33–66% of meibomian gland loss was detected), and grade 3 (>67% of meibomian gland loss was detected) (17). Upper and lower eyelids scores were added to calculate a total meiboscore (0–6) for each eye (18).

Meibomian gland function assessment was conducted in accordance with the recommendations of the International Workshop on Meibomian Gland Dysfunction and TFOS DEWS II (2, 17, 19). For assessing the gland expression, the Meibomian Gland Evaluator (MGE; TearScience, Inc.) was placed in the lower eyelid 2 mm from the root of the eyelash applying constant, gentle pressure for 10–15 s to evaluate meibomian gland secretions by simulating the pressure of a normal blink. And then, the lipid secretion from the meibomian gland orifices was observed through a slit-lamp microscope. Five continuous glands were evaluated, respectively, in the temporal, central, and nasal parts of the lower eyelid, 15 glands in total (20). The meibum expressibility score (MES) was graded by counting the central eight expressed meibomian gland orifices of the lower lid as follows: Grade 0 means all glands are expressible; Grade 1 means 3–4 glands are expressible; Grade 2 means 1–2 glands expressible; Grade 3 means no gland is expressible. The secretion of the meibum quality score (MQS) of each of the 15 glands in the lower eyelid was graded from 0 to 3 as follows: Grade 0, clear; Grade 1, cloudy; Grade 2, cloudy with granular debris; Grade 3, thick like toothpaste or non-expressible glands. The total possible score of both MES and MQS was ranged from 0 to 3 points (2, 17, 19).

Lid margin abnormality score was documented as 0 (absent) or 1 (present) in accordance with the existence of the following four signs: vascular engorgement, plugged meibomian gland orifices, anterior or posterior displacement of the mucocutaneous junction and irregularity of the lid margin. If any of these signs was present, one point was assigned for each item, with a total possible score range of 0–4 points (17).

LipiView® II interferometer was applied to measure LLT of the tear film as described previously: the interferometer converts the specific interference colors into the values of LLT (21). The results of LLT were automatically obtained after the measurement.

### Statistical Analyses

Statistical analyses were performed using Graphpad (version 6.01, San Diego, CA, USA). Comparisons of parameters between the two groups were performed using student-*t* test at baseline and each visit (all parameters are normally distributed). Comparisons of parameters at different visits among the same group were performed with one-way ANOVA. *P* < 0.05 was considered as significant from the statistic perspective.

## Results

### Demographics

Fifty cases were qualified in this study (25 in SS group and 25 in non-SS group). Mean age of the SS patients was 55.41 ± 10.07 and 58.85 ± 15.36 years for non-SS patients. The numbers of males and females were the same in both groups (2 males, 23 females). There was no statistically significant difference of age and dry eye parameters between the two groups (all *P* > 0.05) before punctal obstruction except SPEED scores and CFS scores.

### The Changes of OSDI and SPEED Scores

At the baseline, OSDI score has no difference between the SS group and non-SS group. It was improved at every visit compared with the baseline (all *p* < 0.01) in both SS patients and non-SS patients. However, the patients in SS group had significantly larger OSID scores than that in the non-SS group at 2 weeks, 2 months, and 6 months after punctal plugs insertion (all *p* < 0.01).

SPEED scores in SS group were statistically higher compared with the non-SS group (*p* < 0.05) at the baseline. After the obstruction they were improved in both groups at every visit compared with the baseline (all *p* < 0.05). There had no statistical difference between the two groups at 2 weeks (*P* > 0.05) and the SS group had a significantly larger SPEED scores compared with the non-SS group at 2 months and 6 months (both *p* < 0.01).

The changes of SPEED and OSDI scores in both groups were summarized in Table 1.

**Table 1:** Changes of mean SPEED and OSDI scores in SS and Non-SS group after punctal obstruction.

**Time**	**Pre-punctal obstruction**		**2 weeks after punctal obstruction**		**2 months after punctal obstruction**		**6 months after punctal obstruction**	
	**SS**	**Non-SS**	**SS**	**Non-SS**	**SS**	**Non-SS**	**SS**	**Non-SS**
OSDI score	24.36 ± 6.32	23.08 ± 3.84	16.08 ± 4.65^a^^b^	7.28 ± 1.77^c^	13.32 ± 0.80^a^^b^	3.76 ± 1.23^c^	12.36 ± 2.69^a^^b^	1.96 ± 0.61^c^
SPEED score	12.16 ± 3.31^a^	9.32 ± 1.99	9.28 ± 1.98^b^	7.88 ± 2.13^c^	7.36 ± 1.60^a^^b^	4.48 ± 1.42^c^	8.40 ± 2.18^a^^b^	3.96 ± 1.72^c^

a *p <0.05 in SS vs. Non-SS*.

b *p <0.05 in SS vs. baseline*.

c *p <0.05 in non-SS vs. baseline*.

### Ocular Surface Examination

**(1) TMH**

**Figure 1:**
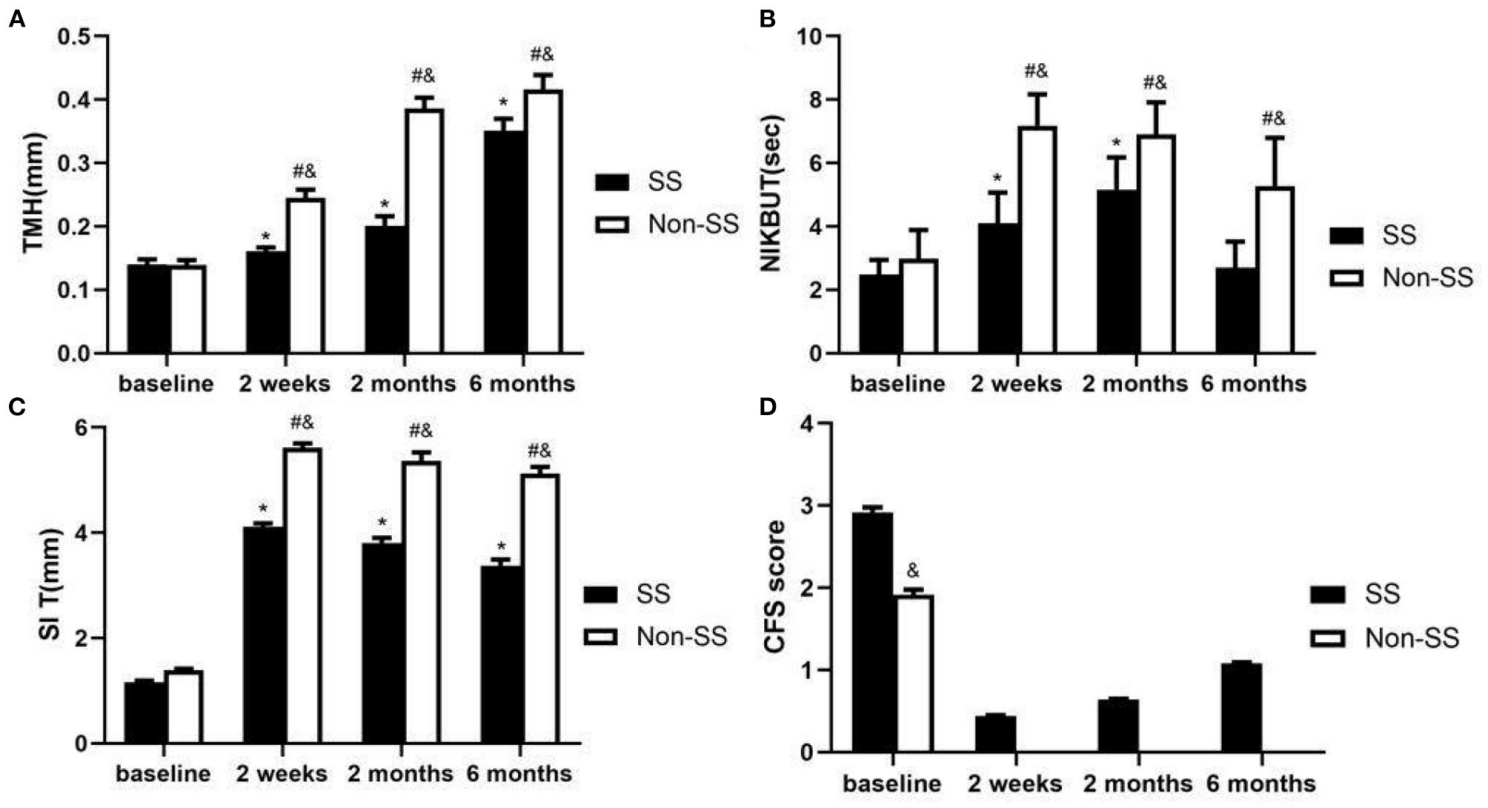
Ocular surface parameter changes were observed in baseline, 2 weeks, 2 and 6 months after punctal plugs insertion. **(A)** Change in TMH (mm). After treatment, a significantly larger TMH was found in non-SS group comparing with SS group at every visit (all *p* < 0.05). In both groups, the TMH was improved at every visit compared to baseline (all *p* < 0.01). **(B)** Change in NIKBUT (sec). Non-SS group had a significantly longer BUT than that in the SS group at every visit. Compared with the baseline, there was statistically significant increase in NIKBUT at every visit in non-SS patients (*p* < 0.01, respectively) and in SS patients NIKBUT was improved at 2 weeks and 2 months (*p* < 0.01, respectively). **(C)** Change in SI T (mm). A significantly larger SI T was found in non-SS group comparing with SS group at every visit (all *p* < 0.01). The SI T score was improved at every visit compared to baseline in both groups (all *p* < 0.01). **(D)** Change in CFS score. There was a statistically significant decrease in the corneal staining score after the treatment, but the SS group showed higher score than the non-SS group (*p* < 0.01) and there was no CFS in non-SS patients. (^&^*p* < 0.05 in SS vs. non-ss, *p < 0.05 in SS vs. baseline, ^#^*p* < 0.05 in non-SS vs. baseline).

The TMH had no difference between the non-SS and SS group at baseline (*P* > 0.05). After punctal plugs insertion, a significantly larger TMH was found in non-SS group comparing with SS group at 2 weeks, 2 months, and 6 months visit (all *p* < 0.05). The TMH improved at every visit compared to baseline (all *p* < 0.01) both in SS group and non-SS group. The highest TMH was noted at 6 months after punctal plugs insertion in both groups (Figure 1A).

**(2) NIKBUT**

At the baseline, there was no statistical difference in NIKBUT between non-SS an SS patients. A statistically longer NIKBUT was found in the non-SS group compared with the SS group at 2 weeks, 2 months, and 6 months after the treatment (*P* < 0.01, respectively). Compared with the baseline, NIKBUT statistically increased in non-SS patients at every visit (*p* < 0.01, respectively), while in SS patients it was improved at 2 weeks and 2 months (*p* < 0.01, respectively) and had no difference at 6 months (*P* > 0.05) (Figure 1B).

**(3) SIT**

Though initial SI T score had no difference between the two groups (*P* > 0.05), a significantly larger SI T was found in non-SS group comparing with SS group at 2 weeks, 2 and 6 months visit after the treatment (all *p* < 0.01). Compared to baseline it improved at every visit both in SS group and non-SS group (all *p* < 0.01). The highest SI T score was noted at 2 weeks after punctal plugs insertion in both groups (Figure 1C).

**(4) CFS**

Comparing with the non-SS group, the SS group had a significantly larger score in CFS at baseline and every visit (all *p* < 0.01). After punctal plugs insertion, there was no CFS in non-SS patients during the follow-up. In SS group, the lowest score was found at 2 weeks, and it slowly increased at 6 months (Figure 1D).

### The Result of Meiboscore, MES, MQS, Lid Margin Abnormality Score and LLT

**(1) Meibomian gland dropout (Meiboscore)**

**Figure 2:**
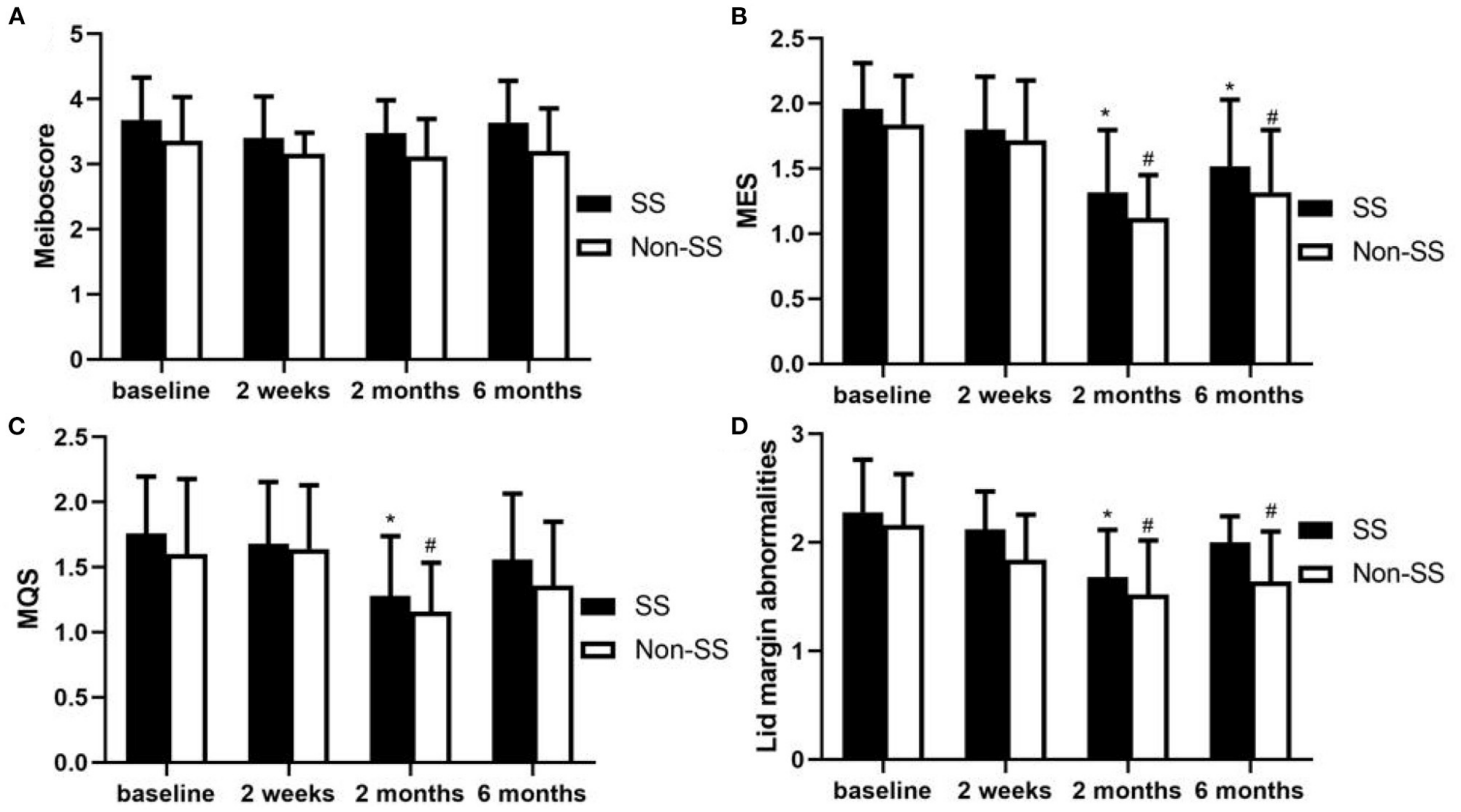
Changes of Meiboscore, MES, MQS and Lid margin abnormality score after punctal plugs insertion. **(A)** There was no statistically significant difference between the two groups in the mean meiboscores at baseline, 2 weeks, 2 and 6 months (*P* > 0.05, respectively). Compared with the baseline, the meiboscores did not change significantly at every visit in both groups. **(B)** The MES had no difference between the two groups at baseline and every visit (all *P* > 0.05). Compared with the baseline, the MES was improved at 2 and 6 months in both groups (*P* < 0.05, respectively). **(C)** There was no obvious difference of MQS between the two groups at baseline and every visit (all *P* > 0.05). Compared with the baseline, the decrease of MQS was statistically significant only at 2 months in both groups. **(D)** There was no statistical difference in the scores of lid margin abnormalities at baseline and every visit between the two groups (*P* > 0.05). After punctal plugs insertion, compared with the baseline, lid margin abnormalities scores were significantly improved in non-SS group at 2 and 6 months (*P* < 0.05, respectively). While SS group showed statistical difference only at 2 months (*P* < 0.05). (*p < 0.05 in SS vs. baseline, ^#^*p* < 0.05 in non-SS vs. baseline).

The meiboscores seemed higher in the SS group than in the non-SS group at baseline, 2 weeks, 2 months, and 6 months, but there was no statistically significant difference between the two groups in the mean meiboscores (*P* > 0.05, respectively). Both in the SS and non-SS dry eye patients, the meiboscores did not change significantly after punctal plugs insertion at 2 weeks, 2, 6 months compared with the baseline (Figure 2A).

**(2) Meibum expressibility score (MES)**

The MES had no difference between the two groups at baseline (*P* > 0.05). After punctal plugs insertion, no statistically difference was found in SS group comparing with non-SS group at 2 weeks, 2 months, and 6 months visit (all *P* > 0.05). Compared with the baseline, the MES was improved in both groups at every visit, but there was no statistical difference at 2 weeks in both groups (*P* > 0.05, respectively). The meibum expressibility seemed to reached a peak at 2 months in both groups (Figure 2B).

**(3) Meibum quality score (MQS)**

There was no significant difference in MQS at baseline between the SS and non-SS group (*P* > 0.05). After the obstruction, the MQS in the SS group looked higher than that in the non-SS group at 2 weeks, 2 months, and 6 months, but no statistical difference was found between the two groups at every visit (*P* > 0.05, respectively). Compared with the baseline, the MQS was decreased in both groups at every visit, but only at 2 months the difference was statistically significant in both groups (*P* < 0.01, respectively) (Figure 2C).

**(4) Lid margin abnormalities**

There was no statistical difference in the scores of lid margin abnormalities at baseline between the two groups (*P* > 0.05). After the obstruction, the scores of lid margin abnormalities in the non-SS group appeared to be lower than that in the SS group during follow-up, but there had no statistical difference between the two groups at each visit (*P* > 0.05, respectively). Compared with the baseline, the scores of lid margin abnormalities were significantly improved in non-SS group at 2 and 6 months (*P* < 0.05, respectively), while SS group showed statistical difference only at 2 months (*P* < 0.05) (Figure 2D).

**(5) LLT**

**Figure 3:**
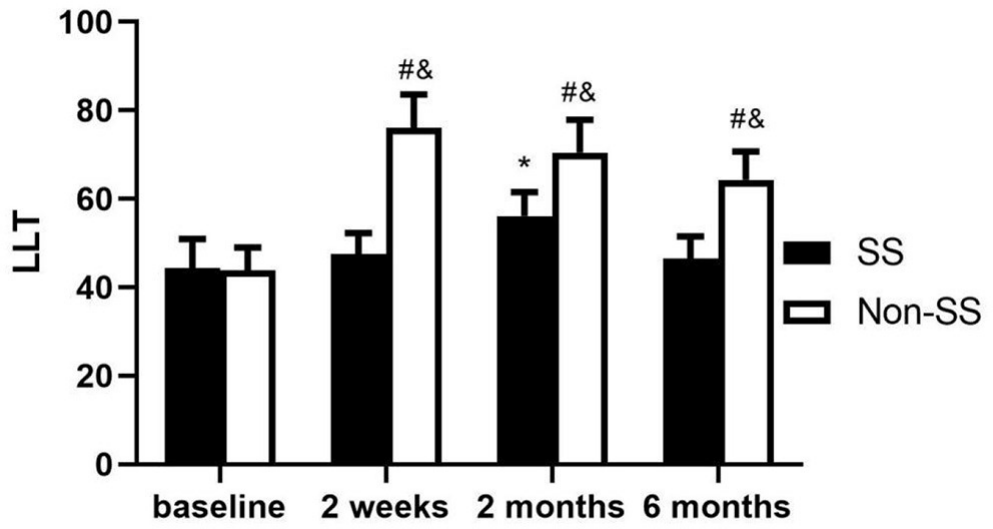
Changes of LLT after punctal plugs insertion. LLT had no difference between the two groups at baseline (*P* > 0.05) and it was significantly larger in the non-SS group than in the SS group at every visit after the treatment (all *p* < 0.05). Compared with the baseline, the increase of LLT in SS group was statistically significant only at 2 months (*p* < 0.05) and in non-SS patients it was greater than the baseline at every visit (*p* < 0.01). (^&^*p* < 0.05 in SS vs. non-ss, *p < 0.05 in SS vs. baseline, ^#^*p* < 0.05 in non-SS vs. baseline).

There was no difference in LLT between the two groups at baseline (*P* > 0.05). At 2 weeks, 2 and 6 months after the obstruction, it was significantly larger in the non-SS group than that in the SS group (all *p* < 0.05). Compared with baseline, LLT increased in SS group after the treatment, but the difference was statistically significant only at 2 months (*p* < 0.05). In non-SS patients, LLT reached its highest level at 2 weeks (*p* < 0.0001) and experienced continuous decrease, although it decreased to some extent at 2 and 6 months, it was still greater than baseline (*p* < 0.05) (Figure 3).

## Discussion

In this study, the clinical features of 25 SS and 25 non-SS ADDE patients before and after punctal plugs insertion were analyzed in dry eye clinical center of the First Affiliated Hospital of Chongqing Medical University in China. We found that both SS and non-SS patients not only had ADDE, but also showed MGD at baseline prior punctal plugs insertion and after punctal obstruction the dry eye parameters were improved in both groups including ocular surface examination, MES, MQS, lid margin abnormalities and LLT, but meiboscores did not change significantly. We also found although the symptoms and some dry eye parameters improved after punctal obstruction in SS patients, they generally did not respond to the treatment as well as non-SS patients during follow-up.

Dry eye is a key finding in SS patients, which has been classified as “aqueous tear deficient dry eye.” However, the possibility of evaporative factors was often overlooked (4, 22). Primarily, SS affects the exocrine glands of mucous membranes such as lacrimal glands, but not the meibomian glands. However, recent studies have found that MGD may also be involved in SS as well (5, 23), and meibomian gland dropout is more severe in SS than in healthy individuals or non-SS dry eye patients (4, 23). This suggests that both aqueous deficiency and tear evaporation were involved in the pathogenesis of dry eye in SS patient (4, 23). The results of our study was consistent with these previous studies. We evaluated a total of 11 dry eye parameters including those related to meibomian glands and the lipid layer of the tear film. Consistent with the reports, our results showed mild to moderate dry eye symptoms at baseline in both groups (5 < average SPEED score <14 and 12 < average OSDI score <32), low BUT (average BUT <5 s), thin LLT (average <60 nm) and MGD as well (lid margin abnormalities score>0, MES <5 and abnormal MQS, etc.) (24). Nowadays, several hypotheses concerning the cause of MGD in SS patients have been raised. First, T-lymphocytes can directly infiltrate and attack the epithelium in the conjunctival area of SS patients (25, 26), probably causing hyperkeratinization of ductal epithelium of the meibomian gland (27). Secondly, female SS patients can experience androgen deficiency (28). As androgen plays an important role in maintaining the normal function of meibomian glands, the decreased level of androgen can then lead to MGD (29). The immunosuppressive action of androgen is well-descried. It was also reported low levels of E2 promote TH1 and cell-mediated response, while high levels of E2 lead to TH2 and humoral response (30). We hypothesize the role of the hormonal affecting the ocular surface in patients with dry eye is mediated by inflammatory cytokines. In the future, further studies will definitely help us in achieving such results.

In our study, a statistically significant decrease in ocular symptom scores (SPEED and OSDI scores) was observed in both SS and non-ss patients after punctal plugs insertion. The SPEED score, which is usually used to assess the frequency and severity of dry eye symptoms over a period of time, was significantly improved from a mean value of 12.16 to 8.40 and from 9.32 to 3.96 between pre-treatment and 6 months after treatment initiation in the SS and non-SS groups, respectively. The cutoff value of the SPEED score to evaluate dry eye disease is 9 (13), with individuals scoring 9 or higher thus complaining of ocular symptoms of dry eye. This means that dry eye symptoms could be relieved in both groups in our study by the plugs for obstruction of the lacrimal drainage system. A similar change was observed in OSDI, SI T, NIBUT and CFS scores at each visit in both groups, except that NIKBUT and CFS score had no significant improvement at 6 months in SS group. Our study confirmed that ocular surface parameters changed from abnormal to normal range or from lower level to higher level after punctal plugs obstruction.

To our knowledge, there are no studies to evaluate the meibomian gland parameters and their changes after punctal plugs insertion in SS dry eye patients. As a chronic, diffuse abnormality of the meibomian glands, both terminal duct obstruction and changes in the glandular secretion may present as typical clinical manifestation in MGD patients (31). Meiboscore, MES, MQS and lid margin abnormality score were often used to evaluate meibomian gland function. In our study, after punctal obstruction, MES decreased at 2 and 6 months of follow-up in both groups, while MQS changed only at 2 months. The lid margin abnormalities were significantly improved in non-SS dry eye patients at 2 and 6 months, and changed in SS group at 6 months, but no significant changes were observed in both groups at 2 weeks. At the same time, there was no significant difference in meiboscores between the SS group and the non-SS group, though meiboscores of the SS group were higher than that of the non-SS group. These results suggest that the function of meibomian glands may be improved to some extent after punctal obstruction, although the number of meibomian glands did not change significantly. However, the reason why punctal plugs can alleviate MGD in SS patients was not investigate in this study. Based on previously published articles, we speculate that inflammation may be a key factor. Inflammation is part of the core pathogenesis of MGD. Increased inflammation is associated with more severe dry eye (32, 33). It can activate expression of pro-inflammatory proteins in the ocular surface (34, 35), which will exacerbate hyperkeratinization of ductal epithelium, aggravate the atrophy of the gland and alter the function of the meibomian gland, finally resulting in MGD (36). Therefore, anti-inflammatory agents have been used chosen to treat MGD (37). Although two recently published papers state that punctal plugs insertion influenced the inflammatory status of ocular surface (38, 39), little evidence can be found concerning the underlying pathogenesis of changes of inflammatory state and tear film stability after puntcal plugs insertion.

The lipid layer in the tear film can prevent tear from evaporating and thus has a protective effect in dry eye (40). It was reported that the lipid layer was thinner and BUT was shorter in SS patients than in non-dry eye controls (4). Previously published study found that LLT was thinner in the MGD patients than control group. Furthermore, LLT was negatively correlated with meibomian gland dropout rate (21). Therefore, LLT may be an indicator of changes in meibomian gland function. That's the reason we measured LLT to evaluate MGD in both groups in this study. In our study, LLT in the non-SS group continued to rise and got a high level at 2 months, while LLT in the SS group only increased at 2 months. This was consistent with the changes of meibomian gland function in both groups. Meanwhile, meiboscores did not change after the treatment, which indicated that the changes in LLT and BUT after punctal plugs insertion might be due to the improvement of the meibomian gland function, instead of the change of the meibomian gland quantity.

In this study, although both SS group and non-SS group obtained improvement in various measured parameters compared with pre-treatment values, it also appeared that non-SS group benefit more from punctal obstruction than SS patients. In non-SS patients, punctal obstruction significantly improved the ocular symptom scores, tear film-related parameters such as BUT, LLT, CFS scores, as well as TMH and SI T compared with SS patients. Even though the meiboscore, MES, MQS and lid margin abnormalities scores did not show a statistically significant difference between the two groups, they were higher or the change lasted for a shorter time in the SS patients compared that in the non-SS patients. We speculated that they were related to more pronounced inflammatory state in SS patients than in non-SS patients. Further analysis is needed to reveal whether inflammatory factors are altered after punctal plugs insertion in SS dry eye patients and whether these changes can alleviate MGD.

However, it should also be noted that there were some limitations to this study. Firstly, this is a prospective study, so selection biases cannot be ignored. Furthermore, there is only a relatively small sample size and short follow-up time. Nevertheless, this study did find that MGD was also present in SS ADDE patients and improved after punctal plugs insertion. Further randomized controlled trials are needed to provide insights into the effect of punctal plugs insertion on the treatment of MGD in ADDE patients.

In conclusion, this study confirmed that both SS and non-SS patients not only had ADDE, but also showed MGD at baseline and after punctal obstruction the dry eye parameters including meibomian gland function improved for at least 6 months in both groups except for meiboscores. In the future, studies will aim to clarify the mechanism of the effect of punctal plugs insertion on the meibomian gland function and how to prolong the therapeutic effect.

## Data Availability Statement

The original contributions presented in the study are included in the article/supplementary material, further inquiries can be directed to the corresponding authors.

## Author Contributions

MX: had full access to all of the data in the study and takes responsibility for the integrity of the data and the accuracy of the data analysis, obtained funding, and supervision. MX and TL: drafting of the manuscript. SL and MX: statistical analysis and administrative, technical, or material support. All authors: critical revision of the manuscript for important intellectual content, concept and design, acquisition, and analysis or interpretation of data.

## Funding

This study was funded by the Chongqing Science and Technology Bureau of China (Grant No. cstc2017jcyjAX0447). The sponsor or funding organization had no role in the design or conduct of this research.

## Conflict of Interest

The authors declare that the research was conducted in the absence of any commercial or financial relationships that could be construed as a potential conflict of interest.

## Publisher's Note

All claims expressed in this article are solely those of the authors and do not necessarily represent those of their affiliated organizations, or those of the publisher, the editors and the reviewers. Any product that may be evaluated in this article, or claim that may be made by its manufacturer, is not guaranteed or endorsed by the publisher.
